# Making organ donation after circulatory death routine: Preserving patienthood and reproducing ways of dying in the intensive care unit

**DOI:** 10.1111/1467-9566.13824

**Published:** 2024-08-16

**Authors:** Jessie Cooper, Zivarna Murphy

**Affiliations:** ^1^ Department of Health Services Research and Management School of Health & Psychological Sciences City St George's, University of London London UK; ^2^ Hull York Medical School University of York York UK

**Keywords:** bioethics, death and dying, end of life care, intensive care, organ donation

## Abstract

Controlled organ donation after circulatory death (DCD) was re‐introduced in the UK in 2008, in efforts to increase rates of organs for transplant. Following reintroduction, there were debates about the ethics of DCD, including whether potential DCD donors receive end‐of‐life care which is in their best interests. Since this time, DCD has become a routine donor pathway in the NHS. In this article, we present findings from an ethnographic study examining the everyday practices of DCD in two English Trusts. Drawing on the concept of death brokering and Bea’s (2020) call to consider organ donation as embedded and routine practice within health care, we look at how DCD is integrated into end‐of‐life care in intensive care units. We show how DCD is made routine at the end‐of‐life via the practices of health professionals who create an active separation between discussions about death and donation; reproduce usual ways of doing things in end‐of‐life care; and respect the distinction between patient/donor, dying and death. In doing so, we argue these function to preserve the patienthood of the potential donor, ensuring DCD operates as an integrated part, and culturally accepted form of, good end‐of‐life care for potential donors, their relatives, and health professionals alike.

## INTRODUCTION

In 2008, as part of efforts to increase the UK’s historically low rates of deceased organ donation, the UK Organ Donor Taskforce (ODT) recommended the need to resolve “outstanding legal, ethical and professional issues” around controlled organ donation after circulatory death (DCD) (Department of Health (DH), 2008b: 9). Controlled DCD refers to donation “from a patient who has died following the (planned) withdrawal of life sustaining treatment” (Dominguez‐Gil et al., 2021, p. 266). It was used in early transplant medicine but was phased out with the introduction of donation after brain death (DBD) in the 1960s, which enabled recovery of perfused organs from brain‐dead patients attached to a ventilator. Until 2008, the practice of DCD was limited to a small number of intensive care units and renal transplant centres in the UK and there were no legal or ethical standards for DCD (Gardiner et al., 2020). The re‐introduction of DCD in the UK and other contexts, like the US and Sweden, led to debates by bioethicists and clinicians about its ethical implications. Among others, these looked at whether potential DCD donors receive appropriate care at the end‐of‐life, since consent and preparations for DCD occur prior to the death of the patient (see Busch & Mjaaland, 2022; Haase et al., 2016; for further insight into ethical issues in DCD). This led to concerns that DCD had the potential to violate a broad interpretation of the dead donor rule: that living patients should not be treated “as though they were dead” for the purposes of organ donation (Gardiner & Sparrow, 2010, p. 17).

Following these debates, there was a rapid policy response, which aimed to standardise the processes of DCD to ensure it could be legally and ethically practiced in the UK (see Cooper, 2017). A key policy was the UK Donation Ethics Committee (UKDEC) and Academy of Medical Royal Colleges (AMRC) ‘*An Ethical Framework for Controlled Donation after Circulatory Death’* (2011) which outlined the ‘principal’ ethical issues related to DCD. One of these is the ‘determination of the potential donor’s best interests’, referring to potential conflicts of interest about decisions around end‐of‐life care and organ donation. These ‘decisions’ include how facilitating DCD may require: “continuing, adjusting or commencing treatments [for example, increasing oxygen concentration and introducing new therapies like inotropic support]; instituting procedures; changing the place of care or other decisions that may have no direct medical benefit to the patient” [for example, in DCD, treatment is usually withdrawn in the anaesthetic room, to allow timely removal of organs in theatre after death] (p.18) but which must be made for the ‘overall benefit of the patient’. In other words, facilitating changes to end‐of‐life care for the purposes of donation are understood to be in the patient’s best interests if their wishes were to be an organ donor.

Since publication of the *ethical framework*, DCD has become a routine donor pathway in the NHS, constituting 43% of deceased donors (NHS Blood and Transplant (NHSBT), 2022), making it important to understand how this form of donation is practiced and experienced by those tasked with carrying it out. In previous work, JC reported on how the routinisation of DCD in the UK has led to new ethical issues related to interactions between organisational timeframes for DCD and (under)resourcing for and de‐prioritisation of donation within the NHS (Cooper, 2021). In this article, we want to shift the focus from categorising ethical issues per se, to examine *how* DCD is integrated as part of end‐of‐life care in the ICU. We present findings from an ethnographic study examining the everyday practices of DCD in two NHS Trusts in England. We show how DCD is made routine at the end‐of‐life through the (extraordinary) practices of health professionals who create an active separation between discussions around death and donation; reproduce usual ways of doing things in end‐of‐life care; and respect the distinction between patient/donor, dying and death. Drawing on social science research on the ‘orchestration’ and brokering of death in medical settings, we argue these practices function to preserve the patienthood of the potential donor, ensuring DCD operates as part and parcel of routine ‘good’ end‐of‐life care, not only for the benefit of potential donors and their relatives, but also for the health professionals tasked with undertaking this form of donation.

### DCD, the ‘good death’, and death brokering

Since the 2008 ODT recommendations, a central remit of the UK’s deceased organ donation strategy has been to make donation a ‘usual not unusual’ event, in part, by incorporating organ donation into end‐of‐life care procedures. In the context of DCD, however, this was understood to lead to potential conflicts of interest. For example, the *ethical framework* outlines how “many of the concerns expressed by physicians and other staff in regard to DCD surround changes to the usual process of caring for dying patients” (UKDEC & AMRC, 2011, p. 46), as detailed earlier. In doing so, DCD essentially ‘reshapes’ end‐of‐life care for the purposes of organ donation (Le Dorze et al., 2022). These concerns are reflected in studies which report on health professional attitudes and perceptions of DCD (Cooper, 2018; Machin et al., 2022; Rodriguez‐Arias et al., 2013). However, the argument in donation and end‐of‐life care policy is that making such changes at the end‐of‐life functions to honour the wishes of the dying, where organ donation is seen as part of creating a ‘good death’. This is reflected in research with donor families, which reports on how relatives find hope and meaning from their loved ones’ organs being used after death (e.g. Jensen, 2016).

This focus on achieving a ‘good death’ is in‐line with UK guidance on end‐of‐life care (Department of Health (DoH) 2008a; Faculty of Intensive Care Medicine, 2019; National Institute for Health and Care Excellence (NICE), 2015), which prioritises treating the individual and those who are important to them with “dignity and respect” (DoH, 2008a, p. 9) and considering their needs and wishes, which, alongside donation, would include ‘cultural, religious, social or spiritual perspectives’ (NICE, 2015). Founded in the hospice and palliative care movement, the ideology of the ‘good death’ is that which attends to the religious, sociocultural and psychological needs of the dying person and their loved ones, alongside ‘aggressive’ symptom management, aiming for death to be socially meaningful and as pain free as possible (Lowrie et al., 2018; Timmermans, 2005). The ‘good death’ is therefore tightly managed and ‘scripted’ by health professionals to ensure control over the process and experience of dying for patients, their relatives and involved staff (Lowrie et al., 2018; Timmermans, 2005).

Work in sociology has long been interested in how death is managed as a highly institutionalised and organised process in medical settings. The seminal ethnographies of Glaser and Strauss (1965) and Sudnow (1967) examined how dying patients were managed in US hospitals, showing death and dying to have a trajectory, which was actively structured and socially produced by the practices of health professionals. More recent studies have examined how technological developments (e.g. the mechanical ventilator, monitoring techniques) have driven new ways of organising death. This includes how health professionals in intensive care contexts enable semblance of a ‘natural’ death through gradual withdrawal of therapies (e.g. Harvey, 1997; Kaufman, 2006; Seymour, 2001) and how, in emergency departments, staff use resuscitation procedures to “soften the abruptness of sudden death”, constructing an acceptable dying trajectory (Brummell et al., 2016, p. 47; Page & Komaromy, 2005; Timmermans, 1998). Stefan Timmermans (2005: 993) has coined such practices a form of “death brokering” referring to the activities of medical professionals to construct individual deaths as ‘culturally meaningful’. Death brokering, according to Timmermans (2005), is a “professional accomplishment” (p.994). It renders the dying process acceptable, achieving a death which is considered ‘good’ and meaningful for all involved, “without disturbing institutional routines” (p.1002), thereby reinforcing and reproducing medical authority over the dying process.

Timmermans contends that the “primary audience” of death brokering are relatives and friends of the dead (2005, p.1002). This reflects social science research on DBD, which has highlighted difficulties experienced by relatives to reconcile the legal diagnosis of (brain) death with how the deceased still appears alive (i.e. warm to touch, colour in their skin, still breathing on a ventilator) (Haddow, 2005; Lock, 2002; Long et al., 2008). Work in sociology and anthropology has described the ways in which brain death is brokered or ‘orchestrated’ by health professionals, to ensure families accept the death as legitimate, enabling them to engage with the request for donation (Cooper & Kierans, 2016; Hadders & Alnaes, 2013; Jensen, 2011; Lock, 2002; Sharp, 2006).

While much focus has been on how brain death is made acceptable for potential DBD families, organ donation also engenders moral and practical problems for health professionals. A small body of research has looked at the ‘transition work’ required by health professionals in seeing a patient as a brain‐dead potential organ donor (Hoeyer et al., 2015; Paul et al., 2014) and described the strategies used by health professionals to deal with moral and ethical issues in managing potential donors (Cooper, 2018; Hoeyer & Jensen, 2012; Sharp, 2006). In relation to DCD, a few scholars have shown how ethical tensions, such as requirements to alter end‐of‐life care, are handled by staff who set ethical limits for themselves (Machin et al., 2022), develop strategies to cope with emotional ambiguities (Le Dorze et al., 2022) and deal with the ethics of DCD as a practical‐organisational problem (Cooper, 2018). Death brokering is therefore highly significant in organ donation, not only to ensure legitimacy of the death for potential donor families, but also render organ donation culturally acceptable for health professional caring for dead and dying potential donors.

However, writing in this journal, Sara Bea (2020: 1936) has pointed out that this work has tended to focus on the “troubling transition from patient to donor”, where ambiguities and dilemmas involved in the shift from patient to donor, from ‘subject to object’, take centre stage. By drawing on science and technology studies (STS) approaches in her ethnographic research on organ donation in a Catalan hospital, Bea looks at how tensions in the donation process (e.g. when health professionals assert that aspects of donor care are not part of their job) are worked through and how organ donation is assembled (i.e. made to work) and potentially disassembled (i.e. does not occur) within a health‐care system that has institutionalised donation as health care. She argues for an alternative perspective, which situates organ donation as integrated and routine practice, carried out as an “ordinary medical activity” (p.1937) within the (public) health‐care system of Spain. With this, she argues that donors remain patients before, during, and after the donation process.

The study on which this article is based was similarly interested in moving away from the often polarised and decontextualised bioethical debates around DCD. The study aimed to understand how ethics emerge and are composed within specific contexts in relation to DCD (i.e. the NHS). This is situated in the tradition of STS approaches to the study of ethics, where ethics are not seen as universal moral norms but need to be investigated as produced and composed within the daily work of health professionals (see Brosnan et al., 2013; Cooper, 2018; Lynch, 2001). In this article, we focus on the particularities of *doing* DCD and ask: what does it mean to care for a potential DCD donor and how is this done within the context of end‐of‐life care within the ICU? Whilst Bea’s (2020) study in Catalonia understands donation as ‘ordinary’ medical activity, we show that, in the UK context, health professionals take extra‐ordinary measures to incorporate DCD as part of routine end‐of‐life care in the ICU, and that they do this as a way to preserve their own (professional and ethical) roles in treating dying patients and maintain the patient status (what we refer to as patienthood) of the potential donor. In so doing, they try to ensure DCD functions as part of good end‐of‐life care for all involved, thereby constructing and maintaining DCD as culturally appropriate at the end of life.

## METHODS

The data in this article is taken from a study which aimed to examine how the process of DCD works in everyday clinical practice to produce insights into its ethical implications. The research study was interested in how health professionals experienced and *did* DCD as part of their work as ICU staff and specialist nurses in organ donation (SN‐ODs) and looking at how DCD was managed at the Trust level. To do this, the project took an ethnographic approach to contextually situate the work of DCD.

The study was granted ethical approval from City, University of London School of Health and Psychological Sciences ethics committee in June 2018 and was given Health Research Authority approval in September 2018 (IRAS project ID: 247,468). Data were collected by JC between October 2018 and June 2019 within two NHS Trusts in England, focussing on one hospital in each Trust (named here as hospital A and B). These hospitals were sampled because they had high rates of DCD, allowing insight into its practice. The study methods involved in‐depth narrative interviews with staff (intensive care consultants, nurses and SNODs) who had experience of DCD, observations of organ donation committee meetings at the Trusts and analysis of Trust documents relating to DCD.

Narrative interviews were used to explore staff practices and experiences around DCD, such as how they cared for and managed potential DCD donors and the challenges involved in DCD and how they dealt with these. Narrative interview questions explore people’s accounts and sense‐making in relation to past events, allowing insight into individual experiences and how these are situated within wider sociocultural contexts (Riessman, 2008). Twenty‐three interviews were conducted with staff across the two hospital sites, including 13 intensive care consultants, three of whom were clinical leads for organ donation (CLODs—ICU consultants given a portion of time to promote organ donation and maximise donation potential within Trusts); two staff nurses and eight SN‐ODs, two of whom were specialist requestors (SN‐ODs who have undergone additional training in communication for the purposes of donation consent). Attempts were also made to interview junior doctors but there were no responses to email requests for interview by doctors below consultant grade. Written consent was taken face‐to‐face prior to each interview. The interviews lasted between 15 and 65 min, were audio recorded and transcribed by two professional transcribers who signed a confidentiality agreement.

Non‐participant observations were conducted of four organ donation committee meetings at the Trusts (two at each) to gain insight into how DCD was discussed at institutional level. Written consent for observations was taken from each committee member at the start of the first meeting at each Trust and was verbally sought at subsequent meetings. Observations were handwritten and later typed. Relevant documents, such as Trust statistics around organ donation and minutes were gathered during and after each meeting. Other Trust documents, such as Standard Operating Procedures (SOPs) and presentations about DCD were also collected as part of the study.

All participants were assigned a number and pseudonym, and other identifying features (such as Trust names, details about donation cases etc.) were anonymised. The interview and observational data were analysed thematically (Braun & Clarke, 2006) to identify and explain typifications across accounts. The documents were used as an aide memoire in the write up of the field notes and to triangulate evidence in relation to Trust responses to issues around DCD, which were relayed in committee meetings and by participants in individual interviews.

## RESULTS

### How DCD gets narrated as routine end‐of‐life practice

When JC first began conducting interviews with intensive care staff and SN‐ODs, she presumed participants would focus on their moral struggles with altering end‐of‐life care processes for the purpose of DCD because that was highlighted as problematic within the bioethics literature. What took her by surprise was the way in which most participants discussed altering end‐of‐life care for DCD as largely unproblematic. Participants’ answers to her questions about their involvement leading up to withdrawal of treatment focused on how timings and decisions altered with DCD. Staff also explained that medications that would be discontinued if a patient was not going for donation would be continued, and some medications would be introduced to stabilise the patient while the donation was being organised, as outlined by one consultant:

If the futility of the treatment in a patient has been established, then you de‐escalate the treatment, that will lead inevitably to death quicker rather than later. What changes when they are deemed to be with DCD […] is then that process [of de‐escalation] gets delayed or stopped altogether. Because then death itself has to be managed. […] So treatments that would have been de‐escalated, removed or limited in any way beforehand are now kept, if not escalated even to allow what now has been established to be a patient’s new wish, which is to donate.

(Hospital A consultant 7)

These decision‐making processes relating to continuing or escalating treatments for the purposes of DCD were largely narrated as procedural rather ethical decisions, and as ones which fulfiled the wish of the potential donor. Many participants took pains to emphasise how DCD did not really differ much from ‘normal’ end‐of‐life care, and one nurse even emphasised how “nothing changes” in relation to end‐of‐life care for potential DCD donors. As one consultant explained:

Consultant So, all the DCD patients should be managed as would any other end‐of‐life care patient. Put on the pathway, until they eventually go to theatre.

JC Does anything get done differently with potential DCD donors around the kind of end‐of‐life care plan?

Consultant No and it shouldn’t be, I mean obviously there will be a lot of activity surrounding the patient because of tissue matching and everything else but, in a way we don’t have a DCD pathway or protocol because the patient should be on the end‐of‐life care plan and shouldn’t be treated any differently to how other dying patients are. (Hospital B, consultant 4)

**FIGURE 1:**
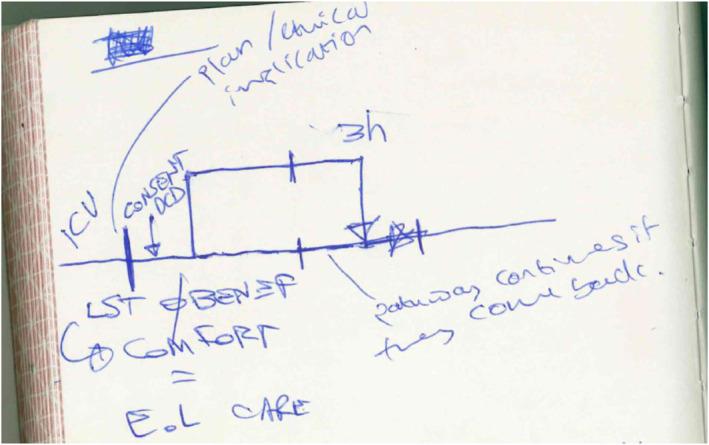
Diagram of DCD in relation to the of end‐of‐life care pathway, drawn by one of the consultant participants in the study. Some annotations are JC’s own based on the consultant’s explanations (plan/ethical implications and ‘pathway continues…’).

DCD was therefore emphasised by staff as being routine in the terms of end‐of‐life care in the ICU. The end‐of‐life care plan mentioned above was illustrated by one of the ICU consultants in Hospital A, who drew this diagram (Figure 1) when asked about the meaning of DCD being part of the typical end‐of‐life care pathway:

In his scribbled diagram, the consultant explained to JC that the lower straight line relates to the non‐donor pathway. The line going off this, which runs up parallel then re‐joins the straight lower line, illustrates what happens when a patient is consented for DCD: they will be re‐routed from this pathway and taken to the anaesthetic room, adjacent to the theatre, to have their treatment withdrawn and to die. If they do not die in the required 3 h for DCD then they will be brought back to the ward, to the straight line, to die. His illustration constructs DCD as a kind of closed circuit, in which its practice never deviates entirely from usual end‐of‐life care.

However, what we went on to realise during analysis is that the described routineness of DCD, as part and parcel of usual end‐of‐life care, was not something that should be taken at face value. Rather than DCD simply aligning with end‐of‐life care in the ICU, we found that health professionals took specific measures to incorporate DCD as part of routine end‐of life care. They did this via practices, which acted to (1) create an active separation between discussions around death and organ donation; (2) reproduce usual ways of doing things in end‐of‐life care in the ICU and (3) respect the distinction between patient/donor, dying and death. In what follows we outline these practices and the ways in which they worked to construct DCD as routine part of end‐of‐life care.

### Creating separation between decisions around treatment withdrawal and DCD

When discussing their experiences around DCD, ICU consultants would often take pains to emphasise how they maintained their role as treating clinicians during the donation process by looking after the best interests of their patients. One area where this was apparent related to clinicians’ accounts of how they created a separation between their discussions with families about the futility of the patient’s situation and the discussions, initiated by SN‐ODs, about organ donation:

I go as far as identifying and referring to the SN‐ODs […]. But that is as far as I go. I do not want to get involved in the organ donation process I find that slightly erm… uncomfortable so I always talk to the families myself first about there is nothing more we can do [related to curative care], I try to avoid being in the room when the SN‐ODs discuss about the patient’s wishes about organ donation.

(Hospital B, Consultant 4)

Such discussions reflected wider policy changes made in the 2008 ODT recommendations for organisational change around organ donation. These included an emphasis on de‐coupling the roles of those who make end‐of‐life decisions (i.e. doctors) and those who ask for and facilitate organ donation (i.e. SN‐ODs). The separation of these roles was reflected on as being important for staff, not only in terms of organising who did what around both DBD and DCD, but also to avoid concerns or accusations around potential conflicts of interest. As one of the consultants put it:

I definitely see that particularly in this day and age where maybe trust in professionals in institutions is a little eroded, that having separation between the clinical side of stuff of us saying this is what we have done, there isn’t any more we can offer and we should stop. Separating that from a discussion about organ donation is probably helpful.

(Hospital A, Consultant 8)

The importance of this separation was made clear in one of the organ donation committee meetings. At this meeting, the donor family representative, whose family member donated their organs at the hospital, was discussing their ideas for having more promotional material for organ donation around the hospital. They suggested it might be a good idea to put posters with stories of transplant recipients in the family rooms of the ICU, so that families in the same situation as they were in could see the positivity of donating. A few of the ICU staff in the room grimaced at this suggestion. Afterwards, one of the consultants told me they felt uncomfortable at this suggestion and did not think it was a good idea, explaining: “we’ve worked really hard at de‐coupling our work from organ donation and we wouldn’t want our work to be misinterpreted”. Whilst the consultant did not provide further detail, it became evident from interviews that this de‐coupling was not just valued for the perceived sake of families but was done to ensure that ICU staff felt comfortable with their role in facilitating donation.

This decoupling was seen as particularly important for DCD, where the separation was perceived to be a challenge. Specifically, since discussions around withdrawal of care and organ donation had to occur while the patient was still alive (different from brain death donation when these discussions will usually happen only once it is suspected that the person has died, or once this has been confirmed with brain death testing), consultants highlighted the difficulty of being able to entirely separate out these two processes:

We are supposed to not allow our decision making [around end of life] to be influenced by the potential for organ donation. So we should be not changing the way we manage the patient, based on the fact that they are a potential organ donor. … and yet we are also supposed to refer 100% of suitable patients for organ donation. So, I don’t know how I can then […] make decisions without that being part of the decision‐making process. I don’t think it is possible to do it. … although that is what we are asked to do.

(Hospital A, Consultant 6)

In practice the imperative for separation between decisions around end of life and organ donation was not seen as tenable by some of the consultants. The doctor above highlights the discomfort this causes, given, what he sees as, the difficulty of making and maintaining this separation. Considering this perceived tension, clinicians described creating an active separation between discussions about withdrawal of treatment and those of organ donation. For some this involved creating a physical separation between themselves and the conversations around donation:

I want to make sure that the family identify my face as a senior medical member of staff who is protecting and looking after the rights of the patient. I don’t want the family to go into donation with any kind of conflict of interest of us withdrawing to extract some organs. So I try to have a final conversation from the medical point of view with the family and to introduce the nurse and then I walk away, literally for the benefit of us all.

(Hospital A, consultant 5)

In emphasising efforts to distance themselves from discussions about donation, the consultants construct and legitimise their role as one which deals with caring for *patients*, not potential donors—the work for which they leave it up to the SN‐ODs.

Whilst this separation helped clinicians maintain their role and avoid perceived conflicts of interest, this sometimes created issues for the work of the SN‐ODs. In particular, the separation was understood to delay the donor referral, even occasionally jeopardising the possibility of a donation:

One of the things that I find most difficult with DCD, from the point of the referral is that we get those referrals quite *late*, so we get them at the point where withdrawal of life‐sustaining treatment conversations have *happened* with the family. I think what a lot of doctors … they like to separate those two things, but what I think they don’t appreciate is that if you tell a family that you're gonna withdraw treatment and you have a time‐frame in mind for that and that’s sort of the next step, families ask the question of what happens next. Um, and because if we’re not on‐board – we work 9 to 5 – we might not be on site when that [futility] conversation happens. So by the time they speak to that family and say someone’s coming to speak to them, it could then be a three‐hour wait because the people who are on call might all be with other people. So for that family, it really prolongs everything because even just to get to the point of having a conversation about donation, as a DCD, it prolongs them.

(Hospital A, SN‐OD 3)

The SN‐OD here articulates her understanding of why clinicians sometimes refer a potential DCD donor to them after the futility conversation—‘they like to separate those two things’. However, these practices of separation were also viewed as disruptive to the donation process by SN‐ODs in that they were perceived to prolong the process for grieving families.

### Reproducing usual ways of doing things: The sedative dilemma

The work by staff to normalise DCD as part of end‐of‐life care was also highlighted in relation to the decision about whether to give potential DCD donors sedatives at the time of their treatment being withdrawn. This related to the common back‐stage (Goffman, 1971 [1959]) understanding in medicine that the administration of sedatives, such as morphine or fentanyl, to ensure that a patient is comfortable and not in pain at the end of life, can also act to speed up the dying process, a phenomenon participants explained was commonly referred to as the ‘double‐effect’ principle (McIntyre, 2019). In the ICU this practice was used, for example, if a patient seemed to be in distress when their breathing tube was removed. Some participants explained their struggles around this practice in general:

The double‐effect principle … with opiates is that if you give a large dose of analgesia like opiate meds […] to relieve the symptoms of distress […] then […] you may have the side effect of reducing their respiratory rate and perhaps hastening their death. […]. We are absolutely not allowed to give medication that we think will hasten the dying process, because that is euthanasia, but there is a grey area there. That is quite a challenge for clinicians, and I think people feel a bit nervous about it. And so I do tend to give at that point, before I extubate someone, a bolus dose (a one off injection) of some fentanyl. And personally I justify that to myself because I say I don’t know if this person is aware or not, I don’t want them to be in any way distressed or uncomfortable, and so I think it is a reasonable thing to do.

(Hospital A, Consultant 4)

While the consultant points out the ‘grey area’ involved in giving opiates as part of end‐of‐life care generally, some staff were explicit in their concerns around how this practice could be perceived in the context of DCD. This related to the fact that DCD donors need to die within a 3‐h window after having their treatment withdrawn for their organs to be useable for donation.^1^ In other words, the practice of giving sedatives to potential DCD donors could be perceived as hastening death to fit into this timeframe, as one SN‐OD explained: “I know that a lot of people do feel conflicted because they don’t wanna start sedative drugs or give *more* because you don’t wanna be seen to *hasten* death” (Hospital A SN‐OD 1). One consultant told me she used to *not* start sedatives at the end of life in DCD because of the potential perception that she would be speeding up the dying process. She explained that she had only changed her approach around this after discussions and reassurance from the medical community that sedatives were appropriate for DCD donors, stating that now: “I don’t see it as something that speeds up the process, but it is just as a comfort measure. I would use it in any other patient I am withdrawing on”.

Significantly, the allocation of labour within ICU meant that it was usually more junior doctors, registrars in training to be intensive care or anaesthetic consultants who would withdraw treatment and make decisions around giving sedatives in the anaesthetic room, where DCD donors are taken to die. Consultants in the study discussed how they would often have to guide and reassure hesitant registrars around what to do in this situation:

They [registrars] are unclear as to exactly what they should be doing at the point when they are in the anaesthetic room and they often ask us questions around … should we be giving them [potential DCD donors] … opioids for pain relief […] my advice is always to say to them, you know, this is a patient that is dying and if you had a patient that was dying on the ward you wouldn’t want them to suffer and if that means there would be a syringe driver perhaps of midazolam and morphine or something on the ward, why would you do that any differently just because it is [potential DCD donor] … and I think some people don’t really think about that it [DCD] is an end of life process, giving them opioids is what you would do normally for a patient that was dying to relieve them from any distress.

(Hospital B, Consultant 2)

The consultant explains how in cases where registrars are uncertain about giving sedatives to potential DCD donors, they ask them to consider what they would do for other (non‐DCD) dying patients—‘you wouldn’t want them to suffer’—to help them see the process as the *same* as for any other end‐of‐life care patient. In this way giving sedatives was normalised and emphasised as an act of *care* for potential donors to prevent suffering at the end of life. By aligning sedative use to patient *care* this justified and legitimised their use on potential DCD donors. The issue then of *how* to discuss the practice of sedation and what to advise more junior doctors to do in relation to potential DCD donors was seen as pressing:

And we had a patient, […] [where] the registrar who was in the anaesthetic room wasn’t sure how much sedation to give. So having some clear guidance on that [is needed] and I think some of the fear of the registrars was that if you gave an opiate [that] would that be seen to hasten the death to achieve the 3 hour threshold […] But I think giving them [junior doctors] some clear guidance on what doses you can give, that you can in fact give it so it is usual end of life care and reinforcing that [is needed].

(Hospital B, consultant 1)

These uncertainties over whether DCD donors should be given sedatives, and if so how much, was highlighted as revealing the need for more concrete guidelines around DCD. This reflects previous work in anthropology about the need for guidelines in dilemmas involving “aggressive” donor management in the context of brain death (Hoeyer & Jensen, 2012). At one of the hospitals, they had begun to take matters into their own hands and, in lieu of national guidelines, were in the process of producing their own localised checklist for clinical staff doing the withdrawal and diagnosing death—as a way of providing more certainty to staff who may have little experience of this process.

### (Re)Creating spaces for dying

The work that went into maintaining good end‐of‐life care in DCD was most strikingly revealed in the spaces in which potential DCD donors were taken to have their treatment withdrawn and to die. The importance of timings in DCD after the person has died—the fact that the surgeon’s knife can go to skin (as expressed by staff) 5 minutes after death is declared—meant that treatment was usually withdrawn on potential donors in the anaesthetic room in the two hospitals (and is usual practice across the NHS). This allowed for the quick transportation of the donor through the double doors into theatre where the retrieval team were waiting to remove the organs. While withdrawing treatment in the anaesthetic room was practically necessary for DCD, participants pointed out that this space is usually used for treating the living. Some felt that this space, in its ‘natural’ form was very clinical, with one consultant pointing out that “it’s not an ideal place of death for a family to remember” (Hospital B, Consultant 2). In theory then, the anaesthetic room was not considered a good space for dying, as one nurse described:

You withdraw a treatment in the anaesthetic room, and…well, it’s not a very nice place to die. [In‐breath] And… […] I think until you're there, in that anaesthetic room, it’s very difficult to…understand it, and it’s a very *cold*, clinical environment; it’s… Critical Care’s also not the nicest place to die, but you can make it a bit more *personal*; you can have people sat with them, and [in‐breath] you've not got an anaesthetic machine and you've not got a team of people going to rush them through.

(Hospital A, nurse 1)

The nurse highlights how the ‘cold’ clinicalness of this space makes it difficult to enable the ‘personal’ touches for dying, possible on the ICU.

As a way of mitigating this, the specialist nurses and some of the clinical leads for organ donation (CLODs) had developed practices to de‐clinicalise this room to make it into a more intimate and appropriate space for dying. This de‐clinicalisation involved making the anaesthetic room more materially suitable for dying, including making the space more *personal* and *personable* to meet the needs of individual families, and physically and spatially demarcating the room as a boundaried place for dying.

Inside the anaesthetic room, the space was materially altered: machines were moved out of the way or covered with sheets, lights were dimmed and electronic tea lights were placed around the room. This was sometimes supplemented with personal objects of the dying patient, such as trinkets and teddy bears. Specialist nurses also tried to make the space as comfortable and personable as possible for the families of potential donors, who would usually be with them for the withdrawal. This included putting tea making facilities in the room and providing the family with blankets to keep them warm:

In the anaesthetic room there’s quite a lot of things, machines, trays, trolleys, so we cover them with the sheet, And I carry with me you know those electrical tealights. […] I put them in the anaesthetic room. It doesn’t really matter to me if they are religious or not, but it is just a nice gesture. Put up tealights, dim the room, tends to be more relaxing if the room is dimmed. […]. But it is a matter of working with the family and giving them that environment as peaceful as it can be for them. […] Prepare seats for them, prepare drinks for them, if they want hot drink or cold drink. […] If the family don’t want to be within that anaesthetic room there is usually a teddy bear or a trinket or, photos and whatnot.

(Hospital B, SN‐OD 3)

The space was also personalised according to the wishes of the family and assumed identity of the donor, for example, one SN‐OD recounted how:

I had a withdrawal and the wife had requested a country singer to be played during the withdrawal – and for him to have a cup of tea next to him, because he used to drink so much tea.

(Hospital A, SN‐OD 1)

In addition, staff often physically demarcated the anaesthetic room as a bounded and boundaried space for dying. This was done by ensuring privacy by locking the doors so people could not easily come in and out; however, since theatre doors do not always have locks this was improvised with tape, twine orwhatever was available. Laminated notices would also be put up asking for the room not to be disturbed. Windows would be blocked with paper, to stop people “peering in”.

Due to time‐pressures of DCD, discussed above, the organ retrieval team wait next door in theatre during the dying process, creating a risk that the private space for dying is disturbed. As a result, the SN‐ODs worked hard to preserve the boundary between dying and donation. This was done by making sure that any donation preparations were done by the retrieval team before the patient was brought into the anaesthetic room. The SN‐ODs also made sure that the retrieval teams did not make noise by limiting communication between the two spaces to text messages or even notes being passed under the door separating the anaesthetic room from the theatre. One SN‐OD explained these practices:

Just before we get the patient into the anaesthetic room you have got all the surgeons scrubbed up and whatnot […]. Then that is [our] avenue to say, ‘be quiet’. Family is going to be next door, no laughing, no doing this [makes an up‐and‐down hitting gesture] with the ice, because they have to break up the blocks of ice [for the organs]. They have to finish that before we get the patient into the anaesthetic room and the family…

(Hospital B, SN‐OD 3)

These activities reveal the work that goes in‐to ensuring the acceptability of the anaesthetic room as a space for dying. In particular, the practices operate to maintain the humanity of the still living donor. While they are set up to die in a space which can facilitate their quick transition to the status of a donor, the activities of staff revealed their treatment as a still‐living patient, with all attempts made to treat them as such and not allowing them to cross over into the category of non‐living donor whilst they are in this space and still alive.

## DISCUSSION AND CONCLUSION

When DCD was reintroduced as a deceased donor pathway in the UK there were concerns that changes to end‐of‐life care to facilitate DCD could threaten the ‘best interests’ of the dying patient. This was due to understanding that these processes have no direct medical benefit for the patient, and, when taken to the extreme, led to concerns that facilitating DCD may violate an interpretation of the dead donor rule. Such concerns led to the production of policies and the *ethical framework* to ensure DCD could be practiced in a way which ‘honours’ the wishes of the dying. These policies and guidelines reassured clinicians that ‘honouring’ someone’s wishes for organ donation (i.e. by making necessary changes to end‐of‐life care for the purposes of donation), is part of the creation of a ‘good death’ for the dying patient. In this article, we have attempted to move away from the decontextualised debates around DCD to try to understand what occurs within the everyday settings in which DCD is practiced. We have taken Bea’s (2020) lead to examine how DCD is integrated as routine end‐of‐life care in the ICU. By looking at the practices and experiences of health professionals around DCD at the end‐of‐life we have also interpreted the wider function of these practices, which we discuss below.

Our findings show that DCD is made routine through practices which function to recreate and reproduce established roles and ways of doing things in end‐of‐life care in the ICU. For example, perceived potential conflicts of interest for treating clinicians were actively avoided via physical separations between futility conversations and those related to organ donation, a finding which is also reflected in a study on DCD in the French context (Le Dorze et al., 2022). However, this separation is not only made possible by policy which recommends such an approach, but also by the existing role hierarchy between treating clinicians and SN‐ODs. The ability of, and importance for senior medical staff to foster this separation also had potential negative implications for the work of the SN‐ODs, whose labour has been shown to often remain hidden and undervalued in organ donation and is sometimes reviled by other medical professionals (Mercado‐Martinez et al., 2013; Sharp, 2006). These relations, which function for the purposes of retaining demarcated roles in organ donation, also therefore reflect the reproduction of the nursing/clinical hierarchies in hospital work, specifically the undervaluing of what is, considered to be, ‘dirty work’ (Bolton, 2005; Douglas, 1966; Miner, 2019).

This reproduction of usual ways of doing things was also highlighted in the material and spatial re‐creation of the anaesthetic room as a suitable environment for dying, and in how uncertainties about giving sedatives are tempered by aligning care of the DCD donor with patient care as part of routine end‐of‐life care processes. DCD is therefore made routine by (re)aligning it with usual ways of treating and caring for dying patients in the ICU, creating an understanding of such practices as *part of*, rather than separated from, good care at the end‐of‐life.

When taken together, these practices show the measures which are taken by staff to ensure that DCD can function as part of routine end‐of‐life care in the ICU. This aligns with, but also moves away from Bea’s (2020) arguments that organ donation is carried out as ‘ordinary’ medical activity, which enacts both as health care and as means to procure organs for transplant. While our findings would agree with her assertion that donors remain patients throughout the process, we contend that, far from being ‘ordinary’ practice, the activities of staff in the NHS is rather extra‐ordinary, in that they work hard to ensure that DCD is incorporated as part of ‘good’ end‐of‐life care in the ICU. In previous work, JC highlighted how donation is de‐prioritised in an NHS subject to austerity (Cooper, 2021) and we could speculate that part of this working hard may be tied to lack of resources and/or priority given for donation in the UK context, in comparison to organ donation in Spain (where Bea’s research was based), which is documented as being well‐resourced and integrated within the health‐care system (Streit et al., 2023).

In addition to understanding *how* DCD is made routine, we can interpret *what* these practices function to achieve in the study context. When simply related to policy, we have demonstrated they function to maintain the dignity of the dying person and that concerns about the broad interpretation of the dead donor rule do not seem to have been upheld. Developing this, we argue that these practices serve to preserve the humanity and social identity of the potential donor, what we have referred to as their patienthood. This argument is influenced by Svendsen’s work  (2011, 2014, following Agamben, 1998) on the socio‐material practices that go into valuing and treating humans and animal neonates as having a qualified biographical life and/or a biological bare life (and the slippage between these categories). In our case what we can understand as the distinction between a dying patient and an organ donor. The construction of DCD as part of routine end‐of‐life care in the ICU thus acts as a form of death brokering (Timmermans, 2005), ensuring the value of the patient as a person (not simply a potential donor) is preserved, meaning DCD functions as an acceptable form of end‐of‐life *care* for potential donors and their relatives.

Whilst Timmermans (2005) contended that death brokering was a “professional accomplishment” (p.994), he highlighted its primary audience to be relatives and friends of the dead. In our study, we have shown that this ‘professional accomplishment’ to integrate DCD as part of routine end‐of‐life care also serves the function of making DCD culturally acceptable for involved health professionals. Previous research studies have described the (ethical and moral) struggles of health professionals with new procedures in organ donation and the practical strategies they adopt to deal with these. This work has highlighted that these strategies are developed at the intersections of professional roles, responsibilities and values and personal morals and conscience (Hoeyer & Jensen, 2012; Le Dorze et al., 2022; Machin et al., 2022; Paul et al., 2014). Whilst health professionals in this study emphasised DCD as being typical or routine in terms of end‐of‐life care, we have shown the work involved in producing this routine‐ness also functions to ensure the preservation of professional roles (ICU doctors and nurses and the SN‐ODs) and the values of staff concerned with creating a ‘good’ and humanising death for dying potential donors.

This study adds to the limited empirical insight into the processes and experiences of DCD since it was re‐implemented. There are a few important limitations to acknowledge. First, the settings were within major trauma centres in England, which had high rates of DCD (known as level one donation centres). The findings may therefore not reflect the practices and experiences within smaller (level two) centres with lower DCD rates, where the integration of DCD into end‐of‐life care may not be so routine. However, with the increasing prevalence of DCD, this article may provide important insights into practices that could be taken up by smaller centres. Second, the participants in the study were mainly ICU consultants and SN‐ODs; there was minimal representation of ICU nurses and no junior clinicians. Further interviews with these groups may have highlighted different experiences, especially as junior doctors were flagged as doing the withdrawal of treatment. Third, the observations were limited to a small number of donation committee meetings, meaning our insight into practices were mainly limited to narrative interview data. It is important that future research gain access to observations of DCD in practice. Finally, this study was focused on staff and has not provided insight into how donor relatives experience the end‐of‐life care of their family member consented to DCD. Future research is needed to understand how these processes are experienced by donor relatives.

What is clear from our findings is that, whilst DCD may now be routine and integrated as part of end‐of‐life care within the NHS, this has been made possible by the hard work of health professionals involved in care and management of dying potential DCD donors. The findings related to the anaesthetic room being made suitable for dying deserve special note and could be used as part of recommendations for good practice, for example, via NHSBT’s education and professional development team. It may also be helpful for the concept of death brokering and our ideas around ‘preserving patienthood’ be used within organ donation training for SN‐ODs and ICU staff as a way of conceptualising what this work does—that is, it attempts to ensure the process of DCD functions as ‘good’ end‐of‐life care for all involved parties.

## AUTHOR CONTRIBUTIONS

**Jessie Cooper**: Conceptualisation (lead); Formal analysis (lead); Funding acquisition (lead); Investigation (lead); Writing – original draft (lead); Writing – review & editing (equal). **Zivarna Murphy**: Formal analysis (supporting); Writing – original draft (supporting); Writing – review & editing (equal).

## CONFLICT OF INTEREST STATEMENT

No conflicts of interest.

## ETHICS STATEMENT

The study was granted ethical approval from City, University of London ethics committee in June 2018 and was given Health Research Authority approval in September 2018 (IRAS project ID: 247468).

## PARTICIPANT CONSENT STATEMENT

All participants in this study gave written, informed consent for their participation and the use of their quotes, on the condition of anonymity and confidentiality.

## Data Availability

The data that support the findings of this study are available on request from the corresponding author. The data are not publicly available due to privacy or ethical restrictions.

## ENDNOTE

^1^
This is due to organ tolerances to warm ischaemia times. Warm ischaemia starts when there is “inadequate oxygenation or perfusion of the organs during withdrawal of treatment or cardiac standstill” (Dunne & Doherty, 2011, p.82). This leads to organ damage, meaning there are limits places on warm ischaemia times following withdrawal of life sustaining treatment in DCD.

## References

[shil13824-bib-0001] Academy of Medical Royal Colleges & UK Donation Ethics Committee . (2011). An ethical framework for controlled donation after circulatory death. Retrieved from http://www.aomrc.org.uk/doc_view/9425‐an‐ethical‐framework‐for‐controlled‐donation‐after‐circulatory‐death

[shil13824-bib-0002] Agamben, G. (1998). Homo sacer. Sovereign power and bare life. Stanford University Press.

[shil13824-bib-0003] Bea, S. (2020). Assembling organ donation: Situating organ donation in hospital practice. Sociology of Health & Illness, 42(8), 1934–1948. 10.1111/1467-9566.13177 32856332

[shil13824-bib-0005] Bolton, S. C. (2005). Women’s work, dirty work: The gynaecology nurse as other. Gender, Work and Organization, 12(2), 169–186. 10.1111/j.1468-0432.2005.00268.x

[shil13824-bib-0006] Braun, V. , & Clarke, V. (2006). Using thematic analysis in psychology. Qualitative Research in Psychology, 3(2), 77–101. 10.1191/1478088706qp063oa

[shil13824-bib-0007] Brosnan, C. , Cribb, A. , Wainwright, S. P. , & Williams, C. (2013). Neuroscientists' everyday experiences of ethics: The interplay of regulatory, professional, personal and tangible ethical spheres. Sociology of Health & Illness, 35(8), 1135–1148. 10.1111/1467-9566.12026 23397962

[shil13824-bib-0008] Brummell, S. P. , Seymour, J. , & Higginbottom, G. (2016). Cardiopulmonary resuscitation decisions in the emergency department: An ethnography of tacit knowledge in practice. Social Science & Medicine, 156, 47–54. 10.1016/j.socscimed.2016.03.022 27017090

[shil13824-bib-0009] Busch, E. J. N. , & Mjaaland, M. T. (2022). Does controlled donation after circulatory death violate the dead donor rule? The American Journal of Bioethics, 23(2), 4–11. 10.1080/15265161.2022.2040646 35238715

[shil13824-bib-0010] Cooper, J. (2017). Problematising the ethics of organ donation after circulatory death in the UK. Critical Public Health, 27(4), 499–505. 10.1080/09581596.2016.1225948

[shil13824-bib-0011] Cooper, J. (2018). Organs and organisations: Situating ethics in organ donation after circulatory death in the UK. Social Science & Medicine, 209, 104–110. 10.1016/j.socscimed.2018.05.042 29852397

[shil13824-bib-0012] Cooper, J. (2021). Time, resourcing and ethics: How the routinisation of organ donation after circulatory death in the NHS has created new ethical issues. Critical Public Health, 33(2), 174–184. 10.1080/09581596.2021.2005241

[shil13824-bib-0013] Cooper, J. , & Kierans, C. (2016). Organ donation, ethnicity and the negotiation of death: Ethnographic insights from the UK. Mortality, 21, 1–18. 10.1080/13576275.2015.1021314

[shil13824-bib-0014] Department of Health . (2008a). End of Life Care Strategy. Promoting high quality care for all adults at the end of life. Retrieved from https://assets.publishing.service.gov.uk/government/uploads/system/uploads/attachment_data/file/136431/End_of_life_strategy.pdf

[shil13824-bib-0015] Department of Health . (2008b). Organs for transplant. HMSO.

[shil13824-bib-0016] Dominguez‐Gil, B. , Ascher, N. , Capron, A. M. , Gardiner, D. , Manara, A. R. , Bernat, J. L. , Minambres, E. , Singh, J. M. , Porte, R. J. , Markmann, J. F. , Dhital, K. , Ledoux, D. , Fondevila, C. , Hosgood, S. , Raemdonck, D. V. , Keshavjee, S. , Dubois, J. , McGee, A. , Henderson, G. V. , & Delmonico, F. L. (2021). Expanding controlled donation after the circulatory determination of death: Statement from an international collaborative. Intensive Care Medicine, 47(3), 265–281. 10.1007/s00134-020-06341-7 33635355 PMC7907666

[shil13824-bib-0017] Douglas, M. (1966). Purity and danger: An analysis of concepts of pollution and taboo. Routledge.

[shil13824-bib-0056] Dunne, K. , & Doherty, P. (2011). Donation after circulatory death. Continuing Education in Anaesthesia Critical Care & Pain, 11(3), 82–86. 10.1093/bjaceaccp/mkr003

[shil13824-bib-0018] Faculty of Intensive Care Medicine . (2019). Care at the end of life: A guide to best practice, discussion and decision‐making in and around critical care. https://www.ficm.ac.uk/sites/ficm/files/documents/2021‐10/ficm‐critical‐condition_0.pdf

[shil13824-bib-0019] Gardiner, D. , Charlesworth, M. , Rubino, A. , & Madden, S. (2020). The rise of organ donation after circulatory death: A narrative review. Anaesthesia, 75(9), 1215–1222. 10.1111/anae.15100 32430909

[shil13824-bib-0020] Gardiner, D. , & Sparrow, R. (2010). Not dead yet: Controlled non‐heart‐beating organ donation, consent, and the dead donor rule. Cambridge Quarterly of Healthcare Ethics, 19(1), 17–26. 10.1017/s0963180109990211 20025799

[shil13824-bib-0021] Glaser, B. G. , & Strauss, A. L. (1965). Awareness of dying. Weidenfeld and Nicolson.

[shil13824-bib-0054] Goffman, E. (1971 [1959]). The presentation of self in everyday life. Penguin.

[shil13824-bib-0022] Haase, B. , Bos, M. , Boffa, C. , Lewis, P. , Rudge, C. , Valero, R. , Wind, T. , & Wright, L. (2016). Ethical, legal, and societal issues and recommendations for controlled and uncontrolled DCD. Transplant International, 29(7), 771–779. 10.1111/tri.12720 26581182

[shil13824-bib-0023] Hadders, H. , & Alnaes, A. H. (2013). Enacting death: Contested practices in the organ donation clinic. Nursing Inquiry, 20(3), 245–255. 10.1111/j.1440-1800.2012.00603.x 22607198

[shil13824-bib-0024] Haddow, G. (2005). The phenomenology of death, embodiment and organ transplantation. Sociology of Health & Illness, 27(1), 92–113. 10.1111/j.1467-9566.2005.00433.x 15762953

[shil13824-bib-0025] Harvey, J. (1997). The technological regulation of death: With reference to the technological regulation of birth. Sociology, 31(4), 719–735. 10.1177/0038038597031004005

[shil13824-bib-0026] Hoeyer, K. L. , & Jensen, A. M. B. (2012). Transgressive ethics: Professional work ethics as a perspective on aggressive organ harvesting. Social Studies of Science, 43(4), 598–618. 10.1177/0306312712460341

[shil13824-bib-0027] Hoeyer, K. L. , Jensen, A. M. B. , & Olejaz, M. (2015). Transplantation as an abstract good: Practising deliberate ignorance in deceased organ donation in Denmark. Sociology of Health & Illness, 37(4), 578–593. 10.1111/1467-9566.12211 25655435

[shil13824-bib-0028] Jensen, A. (2011). Orchestrating an exceptional death: Donor family experiences and organ donation in Denmark. PhD Thesis. https://ifsv.ku.dk/ansatte/?pure=da%2Fpublications%2Forchestrating‐an‐exceptional‐death(a3e1ff6f‐232c‐44cc‐a650‐0424eb475713).html

[shil13824-bib-0029] Jensen, A. (2016). “Make sure somebody will survive from this”: Transformative practices of hope among Danish organ donor families. Medical Anthropology Quarterly, 30(3), 378–394. 10.1111/maq.12278 26768160

[shil13824-bib-0030] Kaufman, S. (2006). And a time to die: How American hospitals shape the end of life. The University of Chicago Press.

[shil13824-bib-0031] Le Dorze, M. , Martouzet, S. , Cassiani‐Ingoni, E. , Roussin, F. , Mebazaa, A. , Morin, L. , & Kentish‐Barnes, N. (2022). “A delicate Balance” – Perceptions and experiences of ICU physicians and nurses regarding controlled donation after circulatory death. A qualitative study. Transplant International, 35, 10648. 10.3389/ti.2022.10648 36148004 PMC9485469

[shil13824-bib-0032] Lock, M. (2002). Twice dead: Organ transplants and the reinvention of death. University of California Press.

[shil13824-bib-0033] Long, T. , Sque, M. , & Addington‐Hall, J. (2008). What does a diagnosis of brain death mean to family members approached about organ donation? Progress in Transplantation, 18(2), 118–125. 10.7182/prtr.18.2.07707n0107q43781 18615977

[shil13824-bib-0034] Lowrie, D. , Ray, R. , Plummer, D. , & Yaa, M. (2018). Exploring the contemporary stage and scripts for the enactment of dying roles: A narrative review of the literature. OMEGA ‐ Journal of Death and Dying, 76(4), 328–350. 10.1177/0030222817696541 29284312

[shil13824-bib-0035] Lynch, M. (2001). The epistemology of epistopics. Science and technology studies as an emergent (non)discipline. In American sociological association science, knowledge and technology section (pp. 2–3). Newsletter, Fall.

[shil13824-bib-0036] Machin, L. , Cooper, J. , Dixon, H. , & Wilkinson, M. (2022). Organ donation in principle and in practice: Health professionals’ troubled consciences. BioSocieties, 17(3), 347–367. 10.1057/s41292-020-00219-z

[shil13824-bib-0037] McIntyre, A. (2019). Doctrine of double effect. In E. N. Zalta (Ed.), The stanford encyclopedia of philosophy. Retrieved from https://plato.stanford.edu/archives/spr2019/entries/double‐effect/

[shil13824-bib-0038] Mercado‐Martinez, F. J. , Diaz‐Medina, B. A. , & Hermandez‐Ibarra, E. (2013). Achievements and barriers in the organ donation process: A critical analysis of donation coordinators’ discourse. Progress in Transplantation, 23(3), 258–264. 10.7182/pit2013410 23996946

[shil13824-bib-0039] Miner, S. A. (2019). Demarcating the dirty work: Canadian fertility professionals’ use of boundary‐work in contentious egg donation. Social Science & Medicine, 221, 19–26. 10.1016/j.socscimed.2018.11.039 30553119

[shil13824-bib-0040] NHS Blood and Transplant . (2022). Organ and tissue donation and transplantation. https://nhsbtdbe.blob.core.windows.net/umbraco‐assets‐corp/27108/activity‐report‐2021‐2022.pdf

[shil13824-bib-0041] NICE . (2015). Care of dying adults in the last days of life. Retrieved from https://www.nice.org.uk/guidance/ng31 26741019

[shil13824-bib-0042] Page, S. , & Komaromy, C. (2005). Professional performance: The case of unexpected and expected deaths. Mortality, 10(4), e307. 10.1080/13576270500321910

[shil13824-bib-0043] Paul, K. T. , Avezaat, C. J. J. , Ijzermans, J. N. , Friele, R. D. , & Bal, R. A. (2014). Organ donation as transition work: Policy discourse and clinical practice in The Netherlands. Health, 18(4), 369–387. 10.1177/1363459313501357 24084010

[shil13824-bib-0044] Reissman, C. K. (2008). Narrative methods for the human sciences. Sage.

[shil13824-bib-0045] Rodriguez‐Arias, D. , Tortosa, J. C. , Burant, C. J. , Aubert, P. , Aulisio, M. P. , & Youngner, S. J. (2013). One or two types of death? Attitudes of health professionals towards brain death and donation after circulatory death in three countries. Medicine, Healthcare & Philosophy, 16(3), 457–467. 10.1007/s11019-011-9369-1 22139386

[shil13824-bib-0046] Seymour, J. (2001). Critical moments: Death and dying in intensive care. Open University Press.

[shil13824-bib-0047] Sharp, L. (2006). Strange harvest: Organ transplants, denatured bodies, and the transformed self. University of California Press.

[shil13824-bib-0055] Streit, S. , Johnston‐Webber, C. , Mah, J. , Prionas, A. , Wharton, G. , Casanova, D. , Mossialos, E. , & Papalois, V. (2023). Ten lessons from the Spanish model of organ donation and transplantation. Transplant International, 36, 11009. 10.3389/ti.2023.11009 37305337 PMC10249502

[shil13824-bib-0048] Sudnow, D. (1967). Passing on: The social organisation of dying. Prentice Hall.

[shil13824-bib-0049] Svendsen, M. N. (2011). Articulating potentiality: Notes on the delineation of the blank figure in human embryonic stem cell research. Cultural Anthropology, 26(3), 414–437. 10.1111/j.1548-1360.2011.01105.x

[shil13824-bib-0050] Svendsen, M. N. (2014). Selective reproduction. Social and temporal imaginaries for negotiating the value of life in human and animal neonates. Medical Anthropology Quarterly, 29(2), 178–195. 10.1111/maq.12149 25359420

[shil13824-bib-0051] Timmermans, S. (1998). Resuscitation technology in the emergency department: Towards a dignified death. Sociology of Health & Illness, 20(2), 144–167. 10.1111/1467-9566.00095

[shil13824-bib-0052] Timmermans, S. (2005). Death brokering: Constructing culturally appropriate deaths. Sociology of Health & Illness, 27(7), 993–1013. 10.1111/j.1467-9566.2005.00467.x 16313526

